# Dimensions of depressive symptoms and cingulate volumes in older adults

**DOI:** 10.1038/tp.2016.49

**Published:** 2016-04-19

**Authors:** M E McLaren, S M Szymkowicz, A O'Shea, A J Woods, S D Anton, V M Dotson

**Affiliations:** 1 Department of Clinical and Health Psychology, University of Florida, Gainesville, FL, USA; 2 Department of Aging & Geriatric Research, University of Florida, Gainesville, FL, USA; 3 Center for Cognitive Aging and Memory, Institute on Aging, Gainesville, FL, USA; 4 Department of Neuroscience, University of Florida, Gainesville, FL, USA

## Abstract

Clinical depression and subthreshold depressive symptoms in older adults have been linked to structural changes in the cingulate gyrus. The cingulate comprises functionally distinct subregions that may have distinct associations with different types, or symptom dimensions, of depression. This study examined the relationship between symptom dimensions of depression and gray matter volumes in the anterior cingulate, posterior cingulate and isthmus of the cingulate in a nonclinical sample. The study included 41 community-dwelling older adults between the ages of 55 and 81. Participants received a structural magnetic resonance imaging scan and completed the Center for Epidemiologic Studies Depression Scale. Subscale scores for depressed mood, somatic symptoms and lack of positive affect were calculated, and Freesurfer was used to extract cingulate gray matter volumes. Regression analyses were conducted to examine the relationship between depressive symptoms and volumes of cingulate subregions while controlling for sex, age and estimated total intracranial volume. Higher scores on the depressed mood subscale were associated with larger volumes in the left posterior cingulate and smaller volumes in the isthmus cingulate. Higher scores on the somatic symptoms subscale were significantly related to smaller volumes in the posterior cingulate. A trend was observed for a positive relationship between higher scores on the lack of positive affect subscale and larger volumes in the anterior cingulate cortex. These results are consistent with previous findings of altered cingulate volumes with increased depressive symptomatology and suggest specific symptom dimensions of depression may differ in their relationship with subregions of the cingulate.

## Introduction

Multiple brain changes have been noted in fronto-limbic pathways in late-life depression, including structural and functional changes in gray matter and increased white matter hyperintensities.^1, 2^ Growing evidence suggests that subthreshold symptoms of depression have similar neural correlates as major depression in older adults, particularly in frontal regions.^2, 3, 4, 5^ The cingulate has been identified as a key area within fronto-limbic networks, in part based on its strong interconnectedness in pathways that are important for mood and emotional processing, including the orbitofrontal cortex, amygdala, hippocampus and striatum.^6^ Converging evidence from structural imaging, functional imaging and neuropathological studies confirm the role of the cingulate in the pathophysiology of depression and in treatment response.^7, 8, 9, 10, 11^

The cingulate is composed of subregions that are dissociable from both a cytoarchitectural and functional standpoint.^12^ Mood disorder research primarily focuses on the anterior cingulate cortex (ACC), which has a role in emotion regulation and reward-based learning, among other functions.^13^ The posterior cingulate cortex (PCC) also has functions relevant to mood, including emotion evaluation, and is important for attention and other cognitive functions.^9, 14, 15^ The isthmus of the cingulate, which connects the PCC to the parahippocampal gyrus, has received less attention in depression research. Nonetheless, the isthmus, along with the ACC and PCC, has been implicated in neuroimaging studies of both major and subthreshold depression, and in both young and older adults.^8, 15, 16, 17, 18, 19, 20, 21^ Structural studies have primarily shown that depression is associated with reduced volumes, thickness and surface area in these regions;^15, 17, 20^ however, depression-related increases have also been reported.^16, 18, 21^

On the basis of the dissociable functions of subregions in the cingulate, it is possible that these regions underlie different types of depressive symptoms. Depression is a heterogeneous construct, with significant variability in symptomatology in individuals with both subthreshold and major depression.^22, 23^ Recent evidence suggests that specific symptom dimensions of depression may be related to unique genetic, physiological and neurological causes, as well as different prognoses and response to treatment.^24, 25, 26^ Relatively few studies have examined neural correlates of symptom dimensions of depression. A small but growing body of work has supported the idea of distinct neural underpinnings of symptom dimensions of depression.^27, 28, 29, 30, 31, 32^ Previous studies have generally used functional imaging measures. Thus, little is known about the relationship between symptom dimensions of depression and brain structure.

The present study aimed to add to this limited literature by examining the relationship of different symptom dimensions of depression, measured by the Center for Epidemiologic Studies Depression Scale (CES-D), with gray matter volumes in subregions of the cingulate in healthy older adults with subthreshold depressive symptoms. The CES-D lends itself to the study of symptom dimensions because of its well-replicated factor structure, which includes depressed mood, somatic symptoms, lack of positive affect and interpersonal difficulties.^33, 34, 35, 36^ There is evidence that these symptom dimensions are differentially associated with cognitive deficits, white matter lesion load and cerebral blood flow.^28, 29, 37^ Depressed mood and somatic symptoms generally have the most salient associations with brain alterations and cognitive deficits in the few existing studies. On the basis of past research, we predicted that higher scores on the depressed mood subscale would be associated with reduced ACC volume, given its role in emotion regulation, and that higher scores on the somatic symptoms subscale would be associated with reduced PCC volume due to its role in attention and other cognitive functions, which in part comprise the CES-D somatic symptoms subscale.

## Materials and Methods

### Participants

Forty-nine healthy, community-dwelling adults aged 55 years and older were recruited. Participants were required to be right-handed, native English speakers with normal or corrected-to-normal vision and 9 or more years of education. Exclusion criteria included evidence of dementia per the Telephone Interview for Cognitive Status,^38^ or self-report of major or unstable medical conditions (for example, uncontrolled hypertension, diabetes, severe cardiac or pulmonary disease, or end-stage kidney or liver disease), neurological disorder (for example, Parkinson's disease, epilepsy, stroke or head injury), learning disorder, current use of antiepileptic or antipsychotic medication, or magnetic resonance imaging contraindications. The protocol was approved by the University of Florida's Health Science Center Institutional Review Board, and all participants gave both written and verbal informed consent to participate in the study. Of the 49 participants recruited for the study, 45 met our criteria, 2 were excluded due to omitting an item on the CES-D and 2 were excluded from analyses because of outlier CES-D score values (>3 s.d.'s above the mean), leaving a total sample size of 41 participants. Two participants scored above 16 on the CES-D, which is the suggested cutoff for clinical depression.^34, 39^ Sample demographic data are presented in Table 1.

### Measurement of depressive symptoms

Depressive symptoms were assessed using the CES-D, a widely used, 20-item self-report measure of depressive symptoms that has been validated in older adults.^34, 40^ The CES-D was selected because it has a well-validated four-factor structure consisting of depressed mood, somatic symptoms, lack of positive affect and interpersonal difficulties subscales.^33^ Questions comprising each subscale are summarized in Table 2. In the current study, 92% of participants had a score of 0 on the interpersonal difficulties subscale, thus scores were not used in our analyses. The depressed mood, somatic symptoms and lack of positive affect subscale scores served as continuous predictors in statistical analyses.

### Imaging procedure

Magnetic resonance imaging data were obtained via a Philips (Amsterdam, The Netherlands) 3-T scanner at the University of Florida's McKnight Brain Institute. An eight-channel head coil was placed over the participant's head as they lay in a supine position on the scanner bed. Foam padding was used to minimize head movement during the scan. Structural images were acquired using a T1-weighted turbo field echo high-resolution three dimensional anatomical scan with 170 1-mm slices in sagittal orientation (repetition time=28.1 ms; echo time=3.7 ms; flip angle=8°).

### Data analyses

Freesurfer image analysis suite (version 5.3) was used for structural data processing. This program is documented and freely available for download online (http://surfer.nmr.mgh.harvard.edu/). Briefly, this processing included skull stripping, automated Talairach transformation, subcortical structure segmentation, intensity inhomogeneity correction, gray/white matter boundary tessellation, topology correction and surface deformation following intensity gradients to ensure optimal gray/white and gray/cerebrospinal fluid border.^41, 42, 43, 44, 45, 46, 47^ The cerebral cortex was parcellated into specific regions with respect to sulcal and gyral structures. Each image was also manually inspected for errors in the automatic program by one of two raters. An interclass correlation coefficient was calculated for volume adjustments using a two-way mixed effects model. Interclass correlation coefficient between raters was extremely high (0.99), likely reflecting the minimal manual adjustments needed following the automatic processing. Gray matter volumes for the ACC, PCC and isthmus of the cingulate were extracted for each hemisphere of the brain. Statistical analyses were conducted using SAS version 9.4 (Cary, NC, USA). Separate regression analyses were conducted for each region with CES-D subscale scores as the predictors and the proportion of regional volume to estimated total intracranial volume as the outcome, controlling for sex and age. The depressed mood, somatic symptoms and lack of positive affect subscales of the CES-D were entered simultaneously in the same regression models to examine the association of each subscale while controlling for other scores. We applied a statistical significance threshold of *α*⩽0.05. Effect sizes are reported as omega squared (*ω*^2^), for which 0.0196 indicates a small effect, 0.059 a medium effect and 0.138 a large effect.^48^ Follow-up analyses were conducted to examine the impact of including a vascular risk covariate in the models (coded 0–2 based on the presence of hypertension, high cholesterol or both) and after applying a square root transformation to address positive skewness of the CES-D subscale scores.

## Results

**Figure 1:**
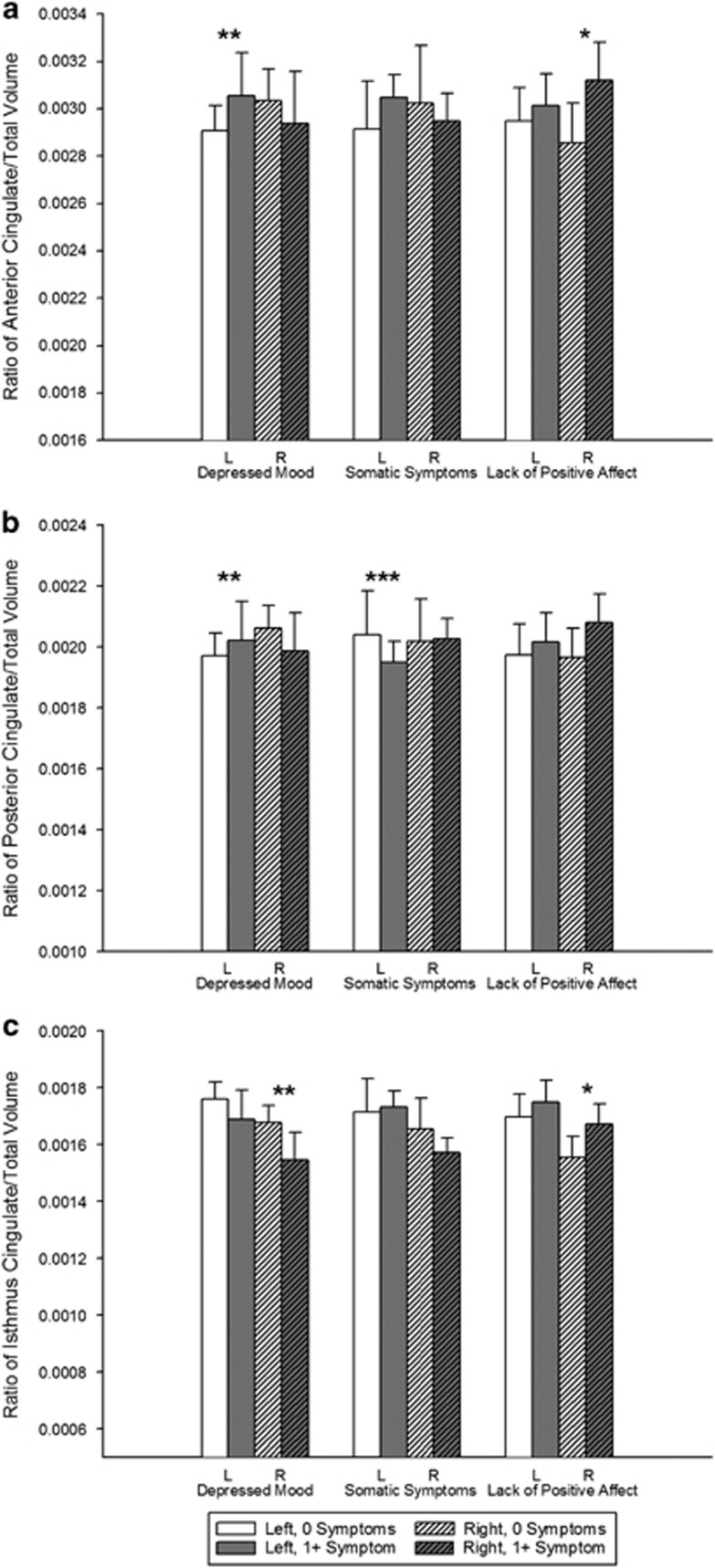
Least-square means showing the ratio of regional to total volume by hemisphere and the Center for Epidemiologic Studies Depression Scale (CES-D) subscale score for the (**a**) anterior cingulate, (**b**) posterior cingulate and (**c**) isthmus cingulate. The CES-D groups are for graphical purposes only; CES-D scores were continuous measures in all analyses. Error bars represent s.e. *represents nonsignificant trends, **P<0.05 and ***P<0.01.

Results of the analyses are summarized in Table 3 and Figure 1. These results are based on analysis of raw data for ease of interpretation. We did not observe meaningful changes to the results when a square root transformation was applied to the CES-D subscale scores, or when a vascular risk covariate was included.

### Anterior cingulate cortex

The CES-D subscale scores were not significantly associated with volumes in the ACC; however, we observed a trend for higher scores on the lack of positive affect subscale to be associated with larger volumes in the right ACC (*F*(5,35)=3.50, *P*=0.070, *ω*^2^=0.060; Figure 1a).

### Posterior cingulate cortex

Higher scores on the depressed mood subscale were associated with larger volumes in the left PCC (*F*(5,35)=5.62, *P*=0.023, *ω*^2^=0.099; Figure 1b). After controlling for age and sex, higher scores on the somatic symptoms subscale were associated with smaller volumes in this region (*F*(5,35)=7.62, *P*=0.009, *ω*^2^=0.142). No effects were found for the right PCC, and lack of positive affect was not associated with PCC volumes.

### Isthmus cingulate

In the analysis of the isthmus of the cingulate (Figure 1c), higher scores on the depressed mood subscale were associated with reduced volumes in the right hemisphere (*F*(5,35)=4.21, *P*=0.048, *ω*^2^=0.075). No effects were found for the left isthmus cingulate, and neither the somatic symptoms nor the lack of positive affect subscales were associated with volumes in this region.

## Discussion

This study examined the relationship between dimensions of depressive symptoms and gray matter volumes in subregions of the cingulate cortex. It was predicted that depressed mood would be associated with decreased ACC volumes, whereas somatic symptoms would be related to reduced PCC volumes. Results partially confirmed the expected pattern of relationships between volumes in cingulate subregions and symptom dimensions of depression; however, we found both positive and negative relationships between depressive symptoms and gray matter volumes in the cingulate.

Consistent with our prediction and with prior research,^49^ higher somatic symptoms of depression were associated with smaller volumes in the left PCC. The somatic symptoms subscale of the CES-D^33^ is somewhat heterogeneous in that it includes items related to traditional somatic complaints (for example, ‘I did not feel like eating'), apathy (for example, ‘I could not get ‘going'') and cognitive difficulty (for example, ‘I had trouble keeping my mind on what I was doing'). The relationship of the somatic subscale with the PCC is not surprising given recent studies that have elucidated roles of the PCC in both mood and cognitive functions, including goal-directed behavior, emotion evaluation, attention, episodic memory and cognitive-affective appraisals.^14, 15, 50, 51^

Our findings suggest that the relationship between depression and PCC volumes is complex. A combination of vegetative, cognitive and apathy symptoms measured by the CES-D somatic symptoms subscale was related to smaller volumes, whereas depressed mood symptoms were related to larger volumes. Consistent with these mixed findings, some structural imaging studies of major and subthreshold depression in older adults report decreased volumes,^15, 17^ whereas others report larger volumes and greater cortical thickness.^16, 18, 21^ In addition, a study of individuals with a familial risk for depression found increased right anterior and posterior cingulate cortical thickness in these individuals.^52^ Current results suggest that conflicting findings regarding the direction of the relationship between depressive symptoms and PCC structure may attributable to different severity of somatic and affective symptoms in previous neuroimaging studies of depression.

Dissociations between symptom dimensions of depression and subregions of the cingulate are also highlighted by the negative relationship of the depressed mood subscale with the isthmus cingulate, in contrast to its positive relationship with the PCC. Less is known about the function of the isthmus cingulate, but there is evidence of its involvement in memory and pain processing,^53^ as well as mood symptoms such as anhedonia and affective flattening.^54^ Alterations in the structure and connectivity of the isthmus have been reported in neuroimaging studies of depression,^16, 19, 20^ consistent with the present results. Our findings highlight the need for focused research on structural and functional alterations of the isthmus of the cingulate in depression, including studies that help to clarify mechanisms by which different symptom dimensions might have opposing relationships with this region.

In addition to the positive relationship between depressed mood and PCC volumes, we found a trend for higher scores on the lack of positive affect subscale to be associated with larger volumes in the ACC. The mechanisms underlying depressive symptom-related volume enlargement are not well understood. There is evidence that the early stages of first-episode depression are associated with increased brain volume due to increased metabolic activity and blood flow, and that over time, mechanisms such as medication use and stress eventually result in decreased volumes.^55^ In addition, studies have shown that early stages of depression may be associated with inflammation that leads to increased cortical thickness,^56, 57, 58^ which is a component of gray matter volume. The current study focused on low levels of depressive symptoms in a nonclinical sample and thus does not directly address this issue. Nonetheless, taken together with the aforementioned findings and previous evidence that subthreshold depression is often a precursor of a major depressive episode,^59, 60^ the present results provide indirect evidence that larger volumes in the ACC may be a prodromal indicator of increased risk for major depression and that different cingulate subregions may be vulnerable to low levels of affective symptoms such as depressed mood and anhedonia in the early course of the disorder.

Overall, results add to a small but growing body of neuroimaging studies examining symptom clusters of depression^27, 28, 29, 61^ by providing evidence that there may be unique relationships between specific symptom dimensions of depression and volumes in subregions of the cingulate. Given the low level of depressive symptoms in this nonclinical sample, further work is needed to clarify whether or not these findings generalize to individuals with clinical depression. Nonetheless, findings related to subthreshold depressive symptoms are important in their own right given evidence that low levels of depressive symptoms are associated with negative sequelae in older adults.^2, 3, 4, 5^ Results should also be considered within the context of the fairly small sample size and lack of information regarding demographic and clinical variables that could impact results, including gender, possibly comorbid anxiety symptoms, antidepressant use, number of previous depressive episodes and age of onset of depressive symptoms. In addition, it should be noted that this is a cross-sectional design, limiting our ability to determine the direction of the relationship between depressive symptoms and brain volumes. Nonetheless, our preliminary findings contribute to the small body of literature related to symptom dimensions of depression. Future studies will incorporate larger samples and use multimodal imaging to elucidate the neural underpinnings of the heterogeneous symptoms of depression. Continued work in this area will increase our understanding of the neurobiology of depression and contribute to the ultimate goal of creating more effective and personalized treatments for those who suffer from the disorder.

## Figures and Tables

**Table 1:** Sample characteristics

	*Mean*	*s.d.*	*Observed range*	*Possible range*
Age (years)	69.68	6.75	55–81	—
Education (years)	15.01	2.56	10–20	—
Gender (% female)	71%	—	—	—
CES-D⩾16 (*N*)	2	—	—	—
				
*CES-D total*	5.88	4.79	0–20	0–60
Depressed mood	1.02	1.67	0–6	0–21
Somatic	2.39	2.18	0–9	0–21
Lack of positive affect	2.34	3.11	0–12	0–12
Interpersonal difficulties	0.10	0.37	0–2	0–6
				
*Medical comorbidities (*N*)*				
Hypertension	12	—	—	—
High cholesterol	17	—	—	—
Arthritis	7	—	—	—

Abbreviation: CES-D, Center for Epidemiologic Studies Depression Scale.

**Table 2:** Item content of the CES-D subscales used in the current study^a^

*Depressed mood*	*Somatic symptoms*	*Lack of positive affect*
I felt that I could not shake off the blues…	I was bothered by things that usually don't bother me	I felt I was just as good as other people
I felt depressed	I did not feel like eating…	I felt hopeful about the future
I thought my life had been a failure	I had trouble keeping my mind on what I was doing	I was happy
I felt fearful	…everything I did was an effort	I enjoyed life
I felt lonely	My sleep was restless	
I had crying spells	I talked less than usual	
I felt sad	I could not get ‘going'	

Abbreviation: CES-D, Center for Epidemiologic Studies Depression Scale.

a The CES-D interpersonal difficulties subscale was not included in the analyses due to the restricted range of scores.

**Table 3:** Results of regression analyses

	*Anterior cingulate cortex*						*Posterior cingulate cortex*						*Isthmus cingulate*					
	*Left*			*Right*			*Left*			*Right*			*Left*			*Right*		
	*b*	*s.e.*	P	*b*	*s.e.*	P	*b*	*s.e.*	P	*b*	*s.e.*	P	*b*	*s.e.*	P	*b*	*s.e.*	P
Mood	0.000010	0.000061	0.864	−0.000095	0.000075	0.211	−0.000097	0.000041	0.023	-0.000050	0.000042	0.250	−0.000038	0.000036	0.301	−0.000070	0.000034	0.048
Somatic	0.000067	0.000044	0.138	0.000057	0.000054	0.306	0.000082	0.000030	0.009	0.000045	0.000031	0.157	0.000019	0.000027	0.483	0.000040	0.000025	0.117
Positive affect	0.000036	0.000026	0.163	0.000059	0.000031	0.070	0.000008	0.000017	0.657	0.000030	0.000018	0.100	0.000005	0.000015	0.725	0.000018	0.000014	0.214

Mood denotes Center for Epidemiologic Studies Depression Scale (CES-D) depressed mood subscale; somatic denotes CES-D somatic symptoms subscale; positive affect denotes CES-D lack of positive affect subscale.

## References

[bib1] Disabato BM, Sheline YI. Biological basis of late life depression. Curr Psychiatry Rep 2012; 14: 273–279. 22562412 10.1007/s11920-012-0279-6 PMC3752388

[bib2] Naismith SL, Norrie LM, Mowszowski L, Hickie IB. The neurobiology of depression in later-life: clinical, neuropsychological, neuroimaging and pathophysiological features. Prog Neurobiol 2012; 98: 99–143. 22609700 10.1016/j.pneurobio.2012.05.009

[bib3] Kumar A, Schweizer E, Zhisong J, Miller D, Bilker W, Swan LL et al. Neuroanatomical substrates of late-life minor depression: a quantitative magnetic resonance imaging study. Arch Neurol 1997; 54: 613–617. 9152118 10.1001/archneur.1997.00550170085018

[bib4] Kumar A, Jin Z, Bilker W, Udupa J, Gottlieb G. Late-onset minor and major depression: early evidence for common neuroanatomical substrates detected by using MRI. Proc Natl Acad Sci USA 1998; 95: 7654–7658. 9636205 10.1073/pnas.95.13.7654 PMC22713

[bib5] Taki Y, Kinomura S, Awata S, Inoue K, Sato K, Ito H et al. Male elderly subthreshold depression patients have smaller volume of medial part of prefrontal cortex and precentral gyrus compared with age-matched normal subjects: a voxel-based morphometry. J Affect Disord 2005; 88: 313–320. 16150493 10.1016/j.jad.2005.08.003

[bib6] Drevets WC, Savitz J, Trimble M. The subgenual anterior cingulate cortex in mood disorders. CNS Spectr 2008; 13: 663–681. 18704022 10.1017/s1092852900013754 PMC2729429

[bib7] Gunning FM, Cheng J, Murphy CF, Kanellopoulos D, Acuna J, Hoptman MJ et al. Anterior cingulate cortical volumes and treatment remission of geriatric depression. Int J Geriatr Psychiatry 2009; 24: 829–836. 19551696 10.1002/gps.2290 PMC2828674

[bib8] Alexopoulos GS, Gunning-Dixon FM, Latoussakis V, Kanellopoulos D, Murphy CF. Anterior cingulate dysfunction in geriatric depression. Int J Geriatr Psychiatry 2008; 23: 347–355. 17979214 10.1002/gps.1939

[bib9] Leech R, Sharp DJ. The role of the posterior cingulate cortex in cognition and disease. Brain 2014; 137: 12–32. 23869106 10.1093/brain/awt162 PMC3891440

[bib10] Hamani C, Mayberg H, Stone S, Laxton A, Haber S, Lozano AM. The subcallosal cingulate gyrus in the context of major depression. Biol Psychiatry 2011; 69: 301–308. 21145043 10.1016/j.biopsych.2010.09.034

[bib11] Sacher J, Neumann J, Funfstuck T, Soliman A, Villringer A, Schroeter ML. Mapping the depressed brain: a meta-analysis of structural and functional alterations in major depressive disorder. J Affect Disord 2012; 140: 142–148. 21890211 10.1016/j.jad.2011.08.001

[bib12] Vogt BA, Finch DM, Olson CR. Functional heterogeneity in cingulate cortex: the anterior executive and posterior evaluative regions. Cereb Cortex 1992; 2: 435–443. 1477524 10.1093/cercor/2.6.435-a

[bib13] Bush G, Luu P, Posner MI. Cognitive and emotional influences in anterior cingulate cortex. Trends Cogn Sci 2000; 4: 215–222. 10827444 10.1016/s1364-6613(00)01483-2

[bib14] Maddock RJ, Garrett AS, Buonocore MH. Posterior cingulate cortex activation by emotional words: fMRI evidence from a valence decision task. Hum Brain Mapp 2003; 18: 30–41. 12454910 10.1002/hbm.10075 PMC6871991

[bib15] Ries ML, Wichmann A, Bendlin BB, Johnson SC. Posterior cingulate and lateral parietal gray matter volume in older adults with depressive symptoms. Brain Imaging Behav 2009; 3: 233–239. 19701486 10.1007/s11682-009-9065-4 PMC2728909

[bib16] Peng D, Shi F, Li G, Fralick D, Shen T, Qiu M et al. Surface vulnerability of cerebral cortex to major depressive disorder. PLoS One 2015; 10: e0120704. 25793287 10.1371/journal.pone.0120704 PMC4368815

[bib17] Lim HK, Jung WS, Ahn KJ, Won WY, Hahn C, Lee SY et al. Regional cortical thickness and subcortical volume changes are associated with cognitive impairments in the drug-naive patients with late-onset depression. Neuropsychopharmacology 2012; 37: 838–849. 22048467 10.1038/npp.2011.264 PMC3260976

[bib18] Li H, Wei D, Sun J, Chen Q, Zhang Q, Qiu J. Brain structural alterations associated with young women with subthreshold depression. Sci Rep 2015; 5: 9707. 25982857 10.1038/srep09707 PMC4434907

[bib19] Korgaonkar MS, Fornito A, Williams LM, Grieve SM. Abnormal structural networks characterize major depressive disorder: a connectome analysis. Biol Psychiatry 2014; 76: 567–574. 24690111 10.1016/j.biopsych.2014.02.018

[bib20] Grieve SM, Korgaonkar MS, Koslow SH, Gordon E, Williams LM. Widespread reductions in gray matter volume in depression. Neuroimage Clin 2013; 3: 332–339. 24273717 10.1016/j.nicl.2013.08.016 PMC3814952

[bib21] Soriano-Mas C, Hernandez-Ribas R, Pujol J, Urretavizcaya M, Deus J, Harrison BJ et al. Cross-sectional and longitudinal assessment of structural brain alterations in melancholic depression. Biol Psychiatry 2011; 69: 318–325. 20875637 10.1016/j.biopsych.2010.07.029

[bib22] Hybels CF, Blazer DG, Pieper CF, Landerman LR, Steffens DC. Profiles of depressive symptoms in older adults diagnosed with major depression: a latent cluster analysis. Am J Geriatr Psychiatry 2009; 17: 387–396. 19390296 10.1097/JGP.0b013e31819431ff PMC2718569

[bib23] Mora PA, Beamon T, Preuitt L, DiBonaventura M, Leventhal EA, Leventhal H. Heterogeneity in depression symptoms and health status among older adults. J Aging Health 2012; 24: 879–896. 22491993 10.1177/0898264312440323

[bib24] NIMHBreaking Ground, Breaking Through: The Strategic Plan for Mood Disorders Research. National Institute of Health: Washington, DC, USA, 2003.

[bib25] Hasler G, Drevets WC, Manji HK, Charney DS. Discovering endophenotypes for major depression. Neuropsychopharmacology 2004; 29: 1765–1781. 15213704 10.1038/sj.npp.1300506

[bib26] Korszun A, Moskvina V, Brewster S, Craddock N, Ferrero F, Gill M et al. Familiality of symptom dimensions in depression. Arch Gen Psychiatry 2004; 61: 468–474. 15123491 10.1001/archpsyc.61.5.468

[bib27] Heinzel A, Grimm S, Beck J, Schuepbach D, Hell D, Boesiger P et al. Segregated neural representation of psychological and somatic-vegetative symptoms in severe major depression. Neurosci Lett 2009; 456: 49–53. 19429132 10.1016/j.neulet.2009.03.097

[bib28] Kirton JW, Resnick SM, Davatzikos C, Kraut MA, Dotson VM. Depressive symptoms, symptom dimensions, and white matter lesion volume in older adults: a longitudinal study. Am J Geriatr Psychiatry 2014; 22: 1469–1477. 24211028 10.1016/j.jagp.2013.10.005 PMC3984387

[bib29] Périco CA-M, Skaf CR, Yamada A, Duran F, Buchpiguel CA, Castro CC et al. Relationship between regional cerebral blood flow and separate symptom clusters of major depression: a single photon emission computed tomography study using statistical parametric mapping. Neurosc Lett 2005; 384: 265–270. 10.1016/j.neulet.2005.04.088 15921853

[bib30] Graff-Guerrero A, González-Olvera J, Mendoza-Espinosa Y, Vaugier V, García-Reyna JC. Correlation between cerebral blood flow and items of the Hamilton Rating Scale for Depression in antidepressant-naive patients. J Affect Disord 2004; 80: 55–63. 15094258 10.1016/S0165-0327(03)00049-1

[bib31] Videbech P, Ravnkilde B, Pedersen TH, Hartvig H, Egander A, Clemmensen K et al. The Danish PET/depression project: clinical symptoms and cerebral blood flow. A regions-of-interest analysis. Acta Psychiatr Scand 2002; 106: 35–44. 12100346 10.1034/j.1600-0447.2002.02245.x

[bib32] Mayberg HS. Limbic-cortical dysregulation: a proposed model of depression. J Neuropsychiatry Clin Neurosci 1997; 9: 471–481. 9276848 10.1176/jnp.9.3.471

[bib33] Shafer AB. Meta-analysis of the factor structures of four depression questionnaires: Beck, CES-D, Hamilton, and Zung. J Clin Psychol 2006; 62: 123–146. 16287149 10.1002/jclp.20213

[bib34] Radloff LS. The CES-D scale: a self-report depression scale for research in the general population. Appli Psychol Meas 1977; 1: 385–401.

[bib35] Helmes E, Nielson WR. An examination of the internal structure of the Center for Epidemiological Studies-Depression Scale in two medical samples. Pers Indiv Differ 1998; 25: 735–743.

[bib36] Carleton RN, Thibodeau MA, Teale MJN, Welch PG, Abrams MP, Robinson T et al. The Center for Epidemiologic Studies Depression Scale: a review with a theoretical and empirical examination of item content and factor structure. PLoS One 2013; 8: e58067. 23469262 10.1371/journal.pone.0058067 PMC3585724

[bib37] Baune BT, Suslow T, Arolt V, Berger K. The relationship between psychological dimensions of depressive symptoms and cognitive functioning in the elderly—the MEMO study. J Psychiatric Res 2007; 41: 247–254. 10.1016/j.jpsychires.2006.06.004 16887147

[bib38] Brandt J, Spencer M, Folstein M. The telephone interview for cognitive status. Neuropsychiatry Neuropsychol Behav Neurol 1988; 1: 111–117.

[bib39] Beekman ATF, Deeg DJH, Van Limbeek J, Braam AW, De Vries MZ, Van Tilburg W. Criterion validity of the Center for Epidemiologic Studies Depression Scale (CES-D): results froma community-based sample of older subjects in the Netherlands. Psychol Med 1997; 27: 231–235. 9122304 10.1017/s0033291796003510

[bib40] Haringsma R, Engels GI, Beekman ATF, Spinhoven P. The criterion validity of the Center for Epidemiological Studies Depression Scale (CES-D) in a sample of self-referred elders with depressive symptomatology. Int J Geriatr Psychiatry 2004; 19: 558–563. 15211536 10.1002/gps.1130

[bib41] Segonne F, Dale A, Busa E, Glessner M, Salat D, Hahn H et al. A hybrid approach to the skull stripping problem in MRI. Neuroimage 2004; 22: 1060–1075. 15219578 10.1016/j.neuroimage.2004.03.032

[bib42] Fischle B, Lui A, Dale A. Automated manifold surgery: constructing gemometrically accurate and topologically correct models of the human cerebral cortex. IEEE Trans Med Imaging 2001; 20: 70–80. 11293693 10.1109/42.906426

[bib43] Fischl B, Dale A. Measuring the thickness of the human cerebral cortex from magnetic resonance images. Proc Natl Acad Sci USA 2000; 91: 11050–11055. 10.1073/pnas.200033797 PMC27146 10984517

[bib44] Fischl B, Salat D, Van der Kouwe A, Makris N, Segonne F, Quinn B et al. Sequence-independent segmentation of magnetic resonance images. Neuroimage 2004; 23: S69–S84. 15501102 10.1016/j.neuroimage.2004.07.016

[bib45] Fischl B, Salat DH, Busa E, Albert M, Dieterich M, Haselgrove C et al. Whole brain segmentation: automated labeling of neuroanatomical structures in the human brain. Neuron 2002; 33: 341–355. 11832223 10.1016/s0896-6273(02)00569-x

[bib46] Sled J, Zijdenbox A, Evans A. A nonparametric method for automatic correction of intensity nonuniformity in MRI data. IEEE Trans Med Imaging 1998; 17: 87–97. 9617910 10.1109/42.668698

[bib47] Dale A, Sereno M. Improved localization of cortical activity by combining EEG and MEG with MRI cortical surface reconstruction: a linear approach. J Cogn Neurosci 1993; 5: 162–176. 23972151 10.1162/jocn.1993.5.2.162

[bib48] Kirk RE. Practical significance: a concept whose time has come. Educ Psychol Meas 1996; 56: 746–759.

[bib49] Walther S, Hugli S, Hofle O, Federspiel A, Horn H, Bracht T et al. Frontal white matter integrity is related to psychomotor retardation in major depression. Neurobiol Dis 2012; 47: 13–19. 22426387 10.1016/j.nbd.2012.03.019

[bib50] Maddock RJ, Garrett AS, Buonocore MH. Remembering familiar people: the posterior cingulate cortex and autobiographical memory retrieval. Neuroscience 2001; 104: 667–676. 11440800 10.1016/s0306-4522(01)00108-7

[bib51] Leech R, Kamourieh S, Beckmann CF, Sharp DJ. Fractionating the default mode network: distinct contributions of the ventral and dorsal posterior cingulate cortex to cognitive control. J Neurosci 2011; 31: 3217–3224. 21368033 10.1523/JNEUROSCI.5626-10.2011 PMC6623935

[bib52] Peterson BS, Weissman MM. A brain-based endophenotype for major depressive disorder. Annu Rev Med 2011; 62: 461–474. 21226617 10.1146/annurev-med-010510-095632 PMC3269401

[bib53] Nielsen FA, Balslev D, Hansen LK. Mining the posterior cingulate: segregation between memory and pain components. Neuroimage 2005; 27: 520–532. 15946864 10.1016/j.neuroimage.2005.04.034

[bib54] Whitford TJ, Lee SW, Oh JS, de Luis-Garcia R, Savadjiev P, Alvarado JL et al. Localized abnormalities in the cingulum bundle in patients with schizophrenia: a Diffusion Tensor tractography study. Neuroimage Clin 2014; 5: 93–99. 25003032 10.1016/j.nicl.2014.06.003 PMC4081981

[bib55] Frodl T, Meisenzahl EM, Zetzsche T, Born C, Jäger M, Groll C et al. Larger amygdala volumes in first depressive episode as compared to recurrent major depression and healthy control subjects. Biol Psychiatry 2003; 53: 338–344. 12586453 10.1016/s0006-3223(02)01474-9

[bib56] Dowlati Y, Herrmann N, Swardfager W, Liu H, Sham L, Reim EK et al. A meta-analysis of cytokines in major depression. Biol Psychiatry 2010; 67: 446–457. 20015486 10.1016/j.biopsych.2009.09.033

[bib57] Liberto CM, Albrecht PJ, Herx LM, Yong VW, Levison SW. Pro-regenerative properties of cytokine-activated astrocytes. J Neurochem 2004; 89: 1092–1100. 15147501 10.1111/j.1471-4159.2004.02420.x

[bib58] Sheline YI, Gado MH, Kraemer HC. Untreated depression and hippocampal volume loss. Am J Psychiatry 2003; 160: 1516–1518. 12900317 10.1176/appi.ajp.160.8.1516

[bib59] Cuijpers P, Smit F. Subthreshold depression as a risk indicator for major depressive disorder: a systematic review of prospective studies. Acta Psychiatr Scand 2004; 109: 325–331. 15049768 10.1111/j.1600-0447.2004.00301.x

[bib60] Lyness JM, Chapman BP, McGriff J, Drayer R, Duberstein PR. One-year outcomes of minor and subsyndromal depression in older primary care patients. Int Psychogeriatr 2009; 21: 60–68. 18786280 10.1017/S1041610208007746 PMC2745243

[bib61] Dotson VM, Davatzikos C, Kraut MA, Resnick SM. Depressive symptoms and brain volumes in older adults: a longitudinal magnetic resonance imaging study. J Psychiatry Neurosci 2009; 34: 367–375. 19721847 PMC2732743

